# Complete genome sequences of nine *Burkholderia pseudomallei* strains

**DOI:** 10.1128/MRA.00400-23

**Published:** 2023-07-25

**Authors:** Takashi Nishida, Yukihiro Hiramatsu, Naoaki Shinzawa, Atsuko Horino, Shigetarou Mori, Yasuhiko Horiguchi

**Affiliations:** 1 Department of Molecular Bacteriology, Research Institute for Microbial Diseases, Osaka University, Osaka, Japan; 2 Department of Parasitology and Tropical Medicine, Graduate School of Medical and Dental Science, Tokyo Medical and Dental University, Tokyo, Japan; 3 Department of Bacteriology II, National Institute of Infectious Diseases, Tokyo, Japan; DOE Joint Genome Institute, Berkeley, California, USA

**Keywords:** *Burkholderia pseudomallei*

## Abstract

We report the complete genome sequences of nine *Burkholderia pseudomallei* strains preserved in research facilities in Japan; GTC3P0019, GTC3P0050, GTC3P0054, GTC3P0254T (type strain), Kanagawa, Tokushima, KM376, KM390, and KM391. The genomic information of these strains may provide references for comparative studies of *B. pseudomallei* pathogenicity.

## ANNOUNCEMENT

*Burkholderia pseudomallei* is the causative agent of melioidosis, a severe disease with a high mortality rate (1, 2). The severity of this disease and the clinical symptoms of patients depend on the route of infection, host immune response, bacterial strains, and the number of bacteria that patients are exposed to. In fact, the 50% lethal dose of the bacteria for mice varies over 1,000-fold between strains (3). However, what bacterial factors determine the differences in virulence between strains is poorly understood.

We sequenced whole genomes of nine *B. pseudomallei* strains from Japanese research facilities (Table 1). After cultivation on a lysogeny broth (LB) agar plate at 37°C for about 38 h, each *B. pseudomallei* strain was recovered from the grown colonies, suspended again in LB to make an optical density at 600 nm of 0.3, and incubated at 37°C for 3 h. The genomic DNA of the cultured bacteria was extracted using a DNeasy Blood and Tissue Kit (Qiagen, Germany). For long-read sequencing, DNA was sheared using a g-TUBE device (Covaris, Woburn, MA, USA), and libraries were prepared from the fragments using a Ligation Sequencing Kit SQK-LSK109 (Oxford Nanopore Technologies, England) and a Native Barcoding Kit (EXP-NBD104 or EXP-NBD114). Long-read sequencing was performed on a MinION sequencer with a FLO-MIN106 flow cell (Oxford Nanopore Technologies, England). The reads were base called using Guppy (Oxford Nanopore Technologies, England) v.4.3.4. or v.6.2.1+6588110 with dna_r9.4.1_450bps_sup.cfg. For short-read sequencing of GTC3P0019, GTC3P0050, GTC3P0054, and GTC3P0254T, the genomic DNA was extracted from bacteria grown from inocula independent of those used for long-read sequencing. Libraries were constructed using a Library Preparation Kit for Illumina (Kapa Biosystems, Wilmington, MA, USA), followed by sequencing on an Illumina MiSeq platform with 251 bp paired-end reads. For short-read sequencing of Kanagawa, Tokushima, KM376, KM390, and KM391, the same DNA used for long-read sequencing was subjected to library preparation. Libraries were constructed using a Nextera DNA Flex Library Preparation Kit (Illumina, San Diego, CA, USA), followed by sequencing on an Illumina NovaSeq platform with 101 bp single-end reads. The quality of the sequencing data was assessed using FastQC v.0.12.1 (4). The total number of reads is shown in Table 1.

**TABLE 1:** Source of the strains and general features of genome sequencing and assembly

Strain	Culture collection^*a*^	Source	Facility^*b*^	Ref.	Read		Coverage	Size (bp)	GC content (%)	No. of CDSs	Accession no.	
					Nanopore (Guppy ver.)	Illumina					DDBJ	DRA (Illumina/ Nanopore)
GTC3P0019	NCTC4845 ATCC15682 GTC3P0019	Monkey, Singapore, 1935	GCMR	(5)	652,893 (6.2.1+6588110)	674,418	676×	Chr1, 3,958,117	68.0	3,467	AP028071	DRR456260/ DRR456269
								Chr2, 3,144,273	68.5	2,459	AP028072	
GTC3P0050	GTC3P0050	Unknown, 1980^*c*^	GCMR		688,562 (6.2.1+6588110)	716,200	681×	Chr1, 4,060,923	67.9	3,563	AP028073	DRR456261/ DRR456270
								Chr2, 3,138,372	68.6	2,473	AP028074	
GTC3P0054	NCTC1688 GTC3P0054	Rat, 1980^*c*^	GCMR	(5)	822,498 (6.2.1+6588110)	619,651	744×	Chr1, 4,050,793	67.9	3,554	AP028075	DRR456262/ DRR456271
								Chr2, 3,198,670	68.5	2,567	AP028076	
GTC3P0254T (type strain)	NCTC12939 ATCC23343 GTC3P0254T	Human, 1980^*c*^	GCMR	(6)	601,165 (6.2.1+6588110)	655,088	537×	Chr1, 3,937,683	68.1	3,432	AP028077	DRR456263/ DRR456272
								Chr2, 3,143,428	68.5	2,462	AP028078	
Kanagawa		Human, Japan, 2010	NIID	(7)	1,074,055 (4.3.4)	8,590,300	926×	Chr1, 4,035,111	68.0	3,536	AP028079	DRR456264/ DRR456273
								Chr2, 3,099,277	68.6	2,433	AP028080	
Tokushima		Human, Japan, 2013	NIID		1,178,142 (4.3.4)	8,258,587	963×	Chr1, 3,997,477	67.9	3,527	AP028081	DRR456265/ DRR456274
								Chr2, 3,175,930	68.5	2,526	AP028082	
KM376		Unknown	IMSUT		721,973 (4.3.4)	8,370,234	549×	Chr1, 3,937,533	68.1	3,435	AP028083	DRR456266/ DRR456275
								Chr2, 3,143,756	68.5	2,472	AP028084	
								Plasmid, 24,809	57.9	27	AP028085	
KM390		Unknown	IMSUT		950,119 (4.3.4)	5,809,045	743×	Chr1, 4,104,708	67.8	3,656	AP028086	DRR456267/ DRR456276
								Chr2, 3,171,481	68.5	2,514	AP028087	
KM391		Unknown	IMSUT		913,723 (4.3.4)	8,777,978	860×	Chr1, 3,957,973	68.0	3,482	AP028088	DRR456268/ DRR456277
								Chr2, 3,144,827	68.5	2,474	AP028089	

^*a*^ NCTC, National Collection of Type Cultures; ATCC, American Type Culture Collection; GTC, Gifu Type Culture Collection.

^*b*^ GCMR, Gifu University Center for Conservation of Microbial Genetic Resource; NIID, The National Institute of Infectious Diseases; IMSUT, The Institute of Medical Science, The University of Tokyo.

^*c*^ Year deposited to GCMR.

*De novo* assembly was conducted using Flye assembler v.2.7-b1585 (8) with sequence reads obtained from the MinION device. The assembled sequences were subsequently polished using Pilon v.1.23 (9) with short reads three times. The assembly revealed one circular chromosome and two contigs in GTC3P0050, four contigs in GTC3P0054, two contigs in Tokushima and KM390, and two circular chromosomes in the other five strains. One plasmid was also assembled in KM376. To verify contig junctions, we synthesized primers (Table 2 at https://doi.org/10.6084/m9.figshare.22632682) that bind to the terminal regions of contigs and performed PCR amplification using KOD FX Neo (TOYOBO, Japan). The PCR products were sequenced on a 3500xL Genetic Analyzer (Applied Biosystems, Foster City, CA, USA), and the sequences of the contig junctions were determined. The genomes were annotated using DFAST v.1.2.18 (10). The results of assembly and annotation are summarized in Table 1. Default parameters were used for all software unless otherwise specified.

## Data Availability

The complete genome sequences were deposited in DDBJ/EMBL/GenBank, and the read data were deposited in the DDBJ Sequence Read Archive. The accession numbers are listed in Table 1.
